# Factors influencing confidence and trust in health professionals: a cross-sectional study of English general practices

**DOI:** 10.3399/BJGP.2025.0154

**Published:** 2026-06-01

**Authors:** Richard Baker, Louis S Levene, Emilie Couchman, Christopher Newby, George K Freeman

**Affiliations:** 1 Division of Public Health and Epidemiology, School of Medical Sciences, University of Leicester, Leicester, UK; 2 General Practitioner, Sarum Health Group, Salisbury, UK; 3 NIHR Clinical Lecturer in General Practice, Academic Unit of Palliative Care | University of Leeds, University of Leeds, Leeds, UK; 4 School of Medicine, University of Nottingham, Nottingham, UK; 5 Department of Primary Care & Public Health, Imperial College London, London, UK

**Keywords:** general practice, trust, triage, remote consultation

## Abstract

**Background:**

Profound changes to English general practice aim to improve access to appointments, however, how these changes might affect patients’ confidence and trust in healthcare professionals is unknown.

**Aim:**

To determine whether appointments that were 1) face to face with any type of healthcare professional, or 2) with GPs in any mode, were associated with variations in confidence and trust in the professional last seen.

**Design and setting:**

A practice-level cross-sectional study of 6196 English general practices was conducted from 2023–2024, using published data.

**Method:**

In a weighted regression model, the outcome was patient-reported confidence and trust in healthcare professionals. The two variables of interest were the percentages of appointments with GPs in any mode or face to face any healthcare professional and the nine covariates were continuity, access, whether patients’ needs were met, the annual appointment rate, practice list size, NHS region, deprivation, and practice populations’ age and ethnicity.

**Results:**

In the model, higher confidence and trust was associated with higher percentages of patients seen by GPs in any mode or face to face any healthcare professional, also with increased continuity, appointment rates, percentages of patients having their needs met, percentages of patients aged >18 years, and percentages of White ethnicity. Confidence and trust was higher in all regions compared with London. Lower confidence and trust was associated with higher deprivation. There was no association with improved access or list size.

**Conclusion:**

This study cannot establish causation but does suggest that strategies to improve access that lower continuity, reduce the percentage of appointments that are with GPs, and increase remote consulting may lead to lower confidence and trust in healthcare professionals.

## How this fits in

A transactional model of general practice is being introduced to improve access that involves triage and increasing percentages of appointments with professionals other than GPs or that are not face to face. Using summary data about almost all English general practices in 2023–2024 with ≥750 patients, the patient-reported levels of confidence and trust from the GP Patient Survey were associated with increased percentages of appointments that were with GPs or were face to face, and with higher continuity, after adjusting for other practice and patient factors. Confidence and trust was lower in practices with fewer appointments per year per patient, fewer patients having their needs met, greater deprivation, fewer patients of White ethnicity, and in practices located in London, compared with other regions of England. Access to general practice needs improving, but the findings of this cross-sectional study suggest that preserving features of relationship-based care is also needed to maintain patients’ trust and confidence in healthcare professionals.

## Introduction

Patient trust in the professionals caring for them is important for optimal healthcare delivery. Patients’ experiences of professionals’ caring, communication, and competence are factors that influence their levels of trust.^1^ Lack of trust can lead to inappropriate underuse or overuse of health care.^2^ It can influence health behaviours, the acceptance of health advice,^3^ adherence,^4^ and health outcomes.^5^

Changes in healthcare provision may undermine trust.^3^ English health care is evolving rapidly. General practice has always relied on a relationship model, characterised by continuity that facilitates familiarity and trust between patient and clinician, and that often improves outcomes.^6,7^ However, propelled by growing workloads, problems with access, and failure to expand the GP workforce, the relationship model is being steadily replaced by a transactional one.^8^ This is characterised by the triage of patients according to assessed need towards a range of healthcare professionals and via different appointment modes (Box 1).

**Box 1.** Comparisons of the relationship and transactional models of general practice

	Relationship model	Transactional model	Consequences
Background	Over repeated consultations, the patient becomes familiar with, and known to, a specific healthcare professional, enabling development of a therapeutic relationship.^6,7,12^ Traditionally, the practitioner has been a general medical practitioner, able to deliver generalist care.	Emerging before but accelerated by the pandemic (Figure 1), the transactional model involves the guiding of patients to a range of healthcare professionals according to structured assessments of their problems. The Modern General Practice Model is an example of this approach, intended to improve patients’ experiences of access.^35,36^	Evidence to compare these two models is relatively limited.
Triage of patients according to assessed need	Less use of triage. Patients needing urgent care seen by familiar professional if possible, but by other staff if not available, but patient choice and preference for continuity determine non-urgent appointments.	Triage, often supported by an online platform, is central to accessing healthcare professionals. Patients routinely directed to different types of staff according to assessed need of presentation.	Patients may have less agency in the transactional model, but the wider range of staff can increase the number of appointments available.
Relationship continuity	Patients usually empowered to consult the same practitioner if they wish.	Selected patients can be prioritised for continuity on clinical grounds, but this is not the default.	Transactional model offers practices greater flexibility in providing appointments. Relationship model facilitates development of continuity.
Appointments with GPs	Appointments are more likely to be with GPs, usually familiar with the patient. One practitioner deals with most problems (generalism).	Appointments are more likely to be with unfamiliar professionals of various professional types and who are less likely to be generalists.	More evidence is needed. Delivering almost all primary care by medical generalists may be inefficient, especially when there is a shortage of GPs. However, the use of a wide variety of non-medical staff may introduce other inefficiencies.
Face-to-face appointments	Most appointments are face to face but remote appointments are used occasionally when convenient and preferred by patients. Patients often enabled to talk with their usual professional in remote consultations (continuity).	Face-to-face appointments usually form the majority of appointments, but a large minority are remote.	Remote appointments can be convenient for practices and patients, but some patients have reported perceived deficiencies in communication, caring, empathy, being listened to and taken seriously in remote appointments.^11,37,38^

**Figure 1.**
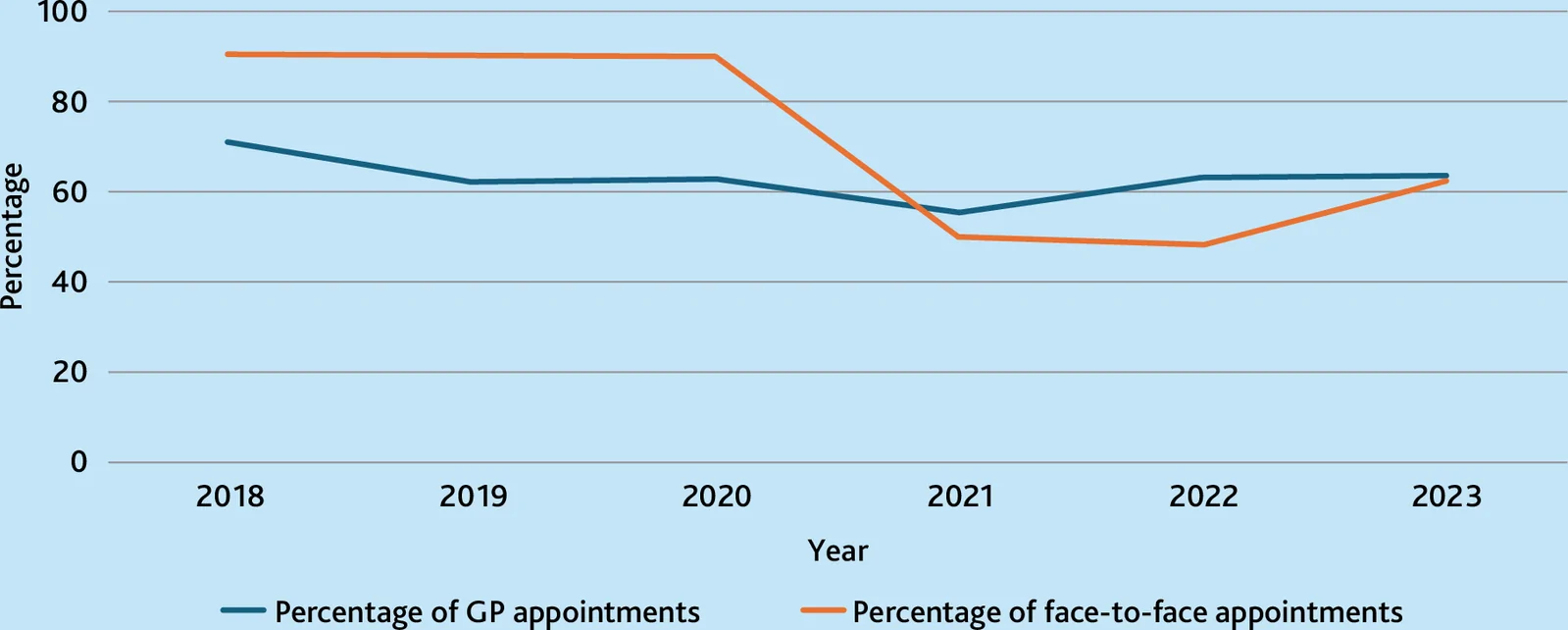
Trends in percentage of appointments with GPs in any mode and percentage of face to face with any healthcare professional from 2018–2023.

In 2018, patients reported 71.0% of appointments as being with GPs in any mode. This fell to 55.5% during the pandemic, before increasing to 63.7% in 2023; 90.7% of all appointments were face to face with any healthcare professional in 2018, 50.0% in the pandemic, and 62.5% in 2023 (Figure 1).^9^ Higher patient satisfaction has been found in practices delivering more GP appointments and more appointments face to face,^10^ but a recent survey revealed many patients lacked confidence about care delivered via telephone or video consultations.^11^

Higher levels of continuity and better ongoing relationships promote increased patient trust in GPs^12–14^ and give patients greater confidence to disclose deeply personal information.^1^ Repeated consultations with the same doctor enable patients to develop trust as their expectations are met.^15^ However, the possible association between levels of patient trust and face-to-face appointments with any type of health professional or appointments with GPs (any mode) has not been investigated. The annual GP Patient Survey (GPPS) in England includes a question on responders’ confidence and trust in the healthcare professional seen at their last appointment,^9^ confidence being defined as an aspect or domain of trust^1,16^ (since the GPPS uses one question to cover confidence and trust and conceptually confidence is a domain of trust, the concept is referred to in the singular). At national level, the percentage of responders who said they definitely had confidence and trust in the professional they last saw or spoke to fell from 69.2% in 2018–2019 to 64.4% in 2023–2024.^9^ Thus, trust was declining while the dominant model of general practice was becoming more transactional.

The study aimed to investigate whether two features of care often associated, but not exclusively, with relationship-based care, were associated with higher levels of trust in healthcare professionals. Specifically, the authors asked whether the percentages of total appointments that were 1) face to face with any healthcare professional, or 2) with a GP in any mode , predicted variations between practices in patient-reported levels of full confidence and trust in the professional they saw at their last appointment.

## Method

This was an ecological cross-sectional study of English general practices in 2023–2024. For 2024, the GPPS was substantially revised and the administration procedure modified.^17^ This created the options of either a cross-sectional study using the most recent data or a longitudinal study using historical data. The former was selected as most likely to be informative about current practice. All data were practice-level published summary statistics. Organisational Data Service (ODS) codes were used to define practices and merge the datasets.^18^

The study population was all practices included in Practice Profiles for 2023–2024,^19^ but eligible practices needed a list size of at least 750 patients and to have data reported in either the Quality and Outcomes Framework (QOF)^20^ or the GPPS,^9^ or both.

### Dependent variable

The dependent variable was each practice’s percentage of responders replying ‘Yes, definitely’ to the 2024 GPPS question: ‘Did you have confidence and trust in the healthcare professional you saw or spoke to?’ (at the last appointment).^9^ The responses, ‘Yes, to some extent’ and ‘No, not at all’, which indicate some or total lack of confidence and trust, were excluded. The revised 2024 question on confidence and trust covered appointments with *any* healthcare professional and *all* modes of consultation.^17^ The GPPS uses weighting strategies to adjust for both practice population characteristics and response rates;^21^ it has been widely used in research studies, producing consistently plausible findings.

### Independent variables

The two variables of interest were percentages of appointments that were face to face (with any healthcare professional) and that were with a GP (any mode). We included nine covariates relating to practice and patient population characteristics expected to influence confidence and trust. Our inclusion criteria were conceptual plausibility, availability of consistently defined data, and absence of strong correlations with other independent variables.

### Appointments

NHS England publishes monthly datasets of appointments provided by general practices, with breakdowns including mode, type of professional, and interval between booking and being seen.^22^ From these data, the mean annual percentage of total appointments that were with GPs (which may be face to face or remote) and the percentage of total appointments that were face to face (face-to-face appointments can be with GPs, nurses, or other professionals) were calculated. Patients’ perceptions of the quality of their appointments may influence confidence and trust in healthcare professionals. From the six questions in the GPPS questionnaire about the most recent appointment, those strongly correlated with continuity or deprivation were excluded, and after reviewing the distribution of responses, the following question was selected: ‘Thinking about the reason for your last appointment, were your needs met?’ The question was selected using the total positive responses to the ‘Yes, definitely’ and ‘Yes, to some extent’ response options (Supplementary Table S1).

### Practice characteristics

Using the appointments datasets, two further variables were added, the percentage of all appointments that were delivered either on the same or following day, and practice list size (mean list size calculated for the year). Practice-level continuity was calculated by multiplying the patient responses to two questions in the 2024 GPPS:^23^ percentage of ‘Yes’ responses to ‘Is there a particular healthcare professional at your GP practice you usually prefer to see or speak to? (This could be a nurse, GP, or other healthcare professional at your practice)’; and the combined percentage of ‘Always or almost always’ and ‘A lot of the time’ responses to ‘How often do you get to see or speak to your preferred healthcare professional when you ask to?’.^14^ Before 2024, these questions referred only to GPs.

To adjust for possible regional variations, the NHS commissioning region in which practices were located was included.^24^ The seven regions were East of England, London, Midlands, North East and Yorkshire, North West, South East, and South West. London was our reference (Table 1).

**Table 1.** Numbers of practices in NHS commissioning region

	NHS commissioning region	Practices (*N*)
1	London	1142
2	South East	796
3	South West	536
4	Midlands	1256
5	North West	947
6	North and East Yorkshire	942
7	East of England	641
	**Total**	6260

### Patient population characteristics

A conceptual framework informed our selection of variables describing practice populations.^25^ The Index of Multiple Deprivation (IMD) was used, last updated in 2019, as a measure of deprivation.^26^ This area-based measure provides a score for each small area, combining indicators from seven domains (income, employment, education, health, crime, housing and services, and environment). Practice Profiles publishes practices’ IMD scores.^19^

Since older patients and those with White ethnicity have been found to be more likely to report confidence and trust,^27^ age (using Practice Profiles) and ethnicity (using the GPPS self-reported results) were adjusted for in practice populations. From the different age bands published by Practice Profiles, the percentage aged <18 years was selected, as this did not correlate strongly with other variables (Supplementary Table S2 ).

The percentage of patients with White ethnicity was chosen as this was the most populous category and did not correlate with other variables (Supplementary Table S3). To represent morbidity, potential variables were assessed, including five QOF prevalence indicators (cancer, hypertension, osteoarthritis, chronic obstructive pulmonary disease [COPD], obesity), two self-reported indicators from the GPPS (unable to work owing to health, smoking), and the annual appointment rate from the appointments data. Annual appointment rate was selected to represent morbidity, as it was the only one not strongly correlated with either IMD, percentage of White ethnicity, or percentage of people aged <18 years (Supplementary Table S3).

### Analysis

The authors checked numeric variables’ distributions and correlations with each other, using Spearman correlation as several ethnicity variables were skewed, to ensure that those variables included in the model were not strongly correlated (coefficient <0.4) (Supplementary Table S4).

For the multivariable analysis, a weighted linear regression was fitted using the response rate to the GPPS, which varies between practices, as the weighting variable. All variables were introduced simultaneously. The model was assessed for collinearity, homogeneity of variance, normality of residuals, influential observations, and a posterior predictive check.

Supplementary analyses included an unweighted regression, models including interactions between GP appointments and face-to-face appointments and between annual appointment rate and continuity, and re-running the weighted model with the ethnicity variable omitted, as this variable’s distribution was slightly skewed (other variables were not, see Table 2). Analyses used R (version 4.4.1).

**Table 2.** Descriptive statistics of numeric variables in the regression model

	*n*	Mean (95% CI)	Median (IQR)
**Dependent variable**			
Percentage of patients definitely having confidence and trust	6253	64.14 (42.01 to 85.23)	64.32 (56.90, 71.77)
**Independent variables**			
Appointments			
Percentage of appointments with a GP	6220	47.99 (21.80 to 72.60)	48.38 (39.04, 57.34)
Percentage of appointments face to face	6220	69.85 (39.17 to 96.01)	70.64 (59.24, 81.37)
Percentage reporting needs were met	6284	90.09 (76.83 to 98.67)	90.89 (86.70-94.41)
Practice characteristics			
List size	6220	9998.83 (2521.26 to 25701.32)	8601.96 (5703.8, 12414.7)
Percentage reporting continuity	6245	14.81 (2.51 to 37.92)	12.82 (8.0, 19.67)
Percentage appointments same day or within 1 day	6220	50.50 (31.59 to 73.62)	49.79 (42.91, 57.44)
Patient population characteristics			
IMD 2019	6348	23.34 (7.21 to 50.50)	21.28 (13.99, 30.26)
Percentage White	6260	76.80 (12.25 to 99.78)	86.87 (64.98, 95.31)
Percentage aged <18 years	6348	21.21 (12.67 to 30.45)	21.03 (18.88, 23.47)
Annual appointment rate	6220	5.68 (3.24 to 9.24)	5.47 (4.62, 6.49)
**Weighting variable**			
Response rate to GPPS %	6260	30.8 (11.81 to 49.38)	31.15 (23.15, 38.34)

IMD = Index of Multiple Deprivation.

## Results

### Study population

In total, 6254 practices with a list size of ≥750 patients and with QOF or GPPS data were included in Practice Profiles for the 2023–2024 year,^19^ complete data being available for 6196 (99.1%).

### Descriptive statistics

Our outcome variable had a normal distribution. A mean of 64.1% (95% confidence interval [CI] = 42.0% to 85.2%) of responders reported that they definitely had confidence and trust in the healthcare professional at their last appointment (Table 2,3). Less than half of all appointments were with GPs in any mode and around two-thirds were face to face with any healthcare professional, but there were also wide variations between practices (Tables 1 and 2). Multiprofessional continuity was below 20% in 70% of practices.

### Multivariable analyses

In our model, higher confidence and trust was associated with higher percentages of appointments with GPs and that were face to face (Table 3). Higher confidence and trust was also associated with higher levels of continuity (Supplementary Figures S1-S3) and of patients’ needs being met at their appointments. Among practice population characteristics, lower confidence and trust was associated with higher IMD 2019 scores and percentages aged <18 years, but higher confidence and trust was associated with higher percentages of White ethnicity, higher annual appointment rates, and location outside London. Practice list size and the percentage of same or next day appointments were not significant. The model’s adjusted *R* squared was 0.622, *F* statistic 640 (*P*<0.001).

**Table 3.** Weighted regression model (6195 general practices)

Variable	Estimate	95% confidence intervals	*P*-value
(intercept)	−37.45	−41.412 to −33.48	<0.001
Appointments			
Percentage appointments with GPs	0.0454	0.031 to 0.060	<0.001
Percentage appointments face to face	0.0134	0.0013 to 0.023	0.030
Percentage needs met	0.981	0.94 to 1.02	<0.001
Practice characteristics			
List size (1000s)	−0.0097	−0.038 to 0.018	0.491
Percentage continuity	0.3388	0.37 to 0.41	<0.001
Percentage appointments on same or within 1 day	0.0022	−0.016 to 0.021 2	0.817
NHS region (reference = London)			
South East	1.218	0.47 to 1.96	0.001
South West	3.217	2.38 to 4.05	<0.001
Midlands	1.225	0.52 to 1.93	<0.001
North West	3.327	2.54 to 4.11	<0.001
North East and Yorkshire	3.251	2.45 to 4.05	<0.001
East of England	0.932	0.14 to 1.73	0.021
Patient population characteristics			
IMD 2019	−0.050	−0.070 to −0.030	<0.001
Percentage White ethnicity	0.0458	0.034 to 0.058	<0.001
Percentage aged <18 years	−0.109	−0.16 to −0.056	<0.001
Annual appointment rate	0.458	0.33 to 0.59	<0.001

IMD = Index of Multiple Deprivation.

### Model checks and supplementary analyses

All the residuals were approximately normally distributed. There was homogeneity of variance. The plots of the residuals versus the predicted values showed no pattern. There was no multicollinearity. These suggest that our model was a suitable fit for the data (Supplementary Figure S4).

In the supplementary analyses, the adjusted *R* square values were slightly lower: 0.607 in the unweighted regression (Supplementary Table S5) and 0.619 in the weighted regression omitting the ethnicity variable (Supplementary Table S6). The predictors were the same except for two variables that no longer predicted in the unweighted model: percentage face-to-face appointments and location in the East of England. The models with interactions did not improve model fit and introduced multicollinearity (Supplementary Table S7).

## Discussion

### Summary

The descriptive statistics showed wide variations in confidence and trust, the percentages seen by GPs, and the percentages seen face to face. To answer our research question, higher levels of confidence and trust in professionals were associated with more patients seen by GPs, and to a lesser extent, seen face to face. Additionally, as expected, higher confidence and trust was associated with higher levels of continuity and of the patient’s needs being met at consultations. In contrast, higher confidence and trust was not associated with higher percentages of same or next day appointments, perhaps because access is more pertinent to patients’ trust in practices than in healthcare professionals themselves.^1^

To provide an indication of the relevance of the associations, the authors converted the regression coefficients into the associated changes (Supplementary Table S8) (see limitations below). Of the factors more common to relationship-based care, continuity had the strongest association, followed by appointments with GPs in any mode then by face-to-face appointments with any healthcare professional. If these three variables are viewed collectively as typifying relationship-based care, the strength of the associations do indicate their importance to maintaining trust in healthcare professionals.

The findings also highlight inequities between patient groups: patients in practices with more deprived patients, more with non-White ethnicity, and in London all reported lower confidence and trust.

### Strengths and limitations

Our study covered 99% of active English general practices. The multivariable model included most of the variables expected to predict levels of confidence and trust. Although there was no specific measurement of morbidity, other variables, such as IMD 2019, annual appointment rate, age and ethnicity, either incorporate measures of morbidity or reflect it. The GPPS carefully adjusts for low response rates^21^ and is widely used in research studies.^28^

The GPPS changed the definitions of some questions in 2024, preventing a longitudinal analysis that included up-to-date data. Ecological studies cannot determine causation, nor can it be assumed that practice-level associations apply at the patient level.

### Comparison with existing literature

To our knowledge, this is the first study to report associations between percentages of GP appointment in any mode and face-to-face appointments with any healthcare professional and levels of confidence and trust in the professional last seen. The findings are consistent with other studies that raised concerns about the fragmentation of care,^29^ and reported lower patient satisfaction in practices where smaller proportions of consultations were face to face with either a GP or any healthcare professional and same day appointments were prioritised.^30^ A recent review concluded that patients want access to involve choice and, ideally, an appointment with a specific GP or clinician.^31^ Other research has shown that satisfaction is higher in practices with more GPs per unit of population and with more face-to-face appointments,^10^ that around only half of patients have confidence in remote appointments,^11^ and that patients consulting paramedics in general practice reported lower confidence compared with those consulting GPs.^32^ There is, therefore, a growing body of evidence suggesting the need for caution when reducing the percentages of appointments that are with GPs in any mode or face to face with any healthcare professional.

### Implications for research and practice

Although from a cross-sectional study, our findings question the wisdom of adopting largely transactional models of general practice to replace relationship models. In improving access, strategies should be avoided that risk weakening patients’ confidence and trust in their healthcare professionals. Easy, equitable access, and relationship-based care are both needed, but achieving this requires adequate resourcing of general practice.^33^ Declining trust in healthcare professionals matters because trust influences patients’ use of resources, their health behaviours, and their outcomes.^1–5^ Research is needed to assess the impact of declining confidence and trust on patient acceptance of clinical advice, such as on the increasing use of emergency departments. If the transactional model is to become the norm, interventions to preserve or improve confidence and trust within it should be developed and evaluated. Research is also needed to better understand the explanations for and the effects of lower confidence and trust in practices with more patients from socioeconomically deprived or ethnic minority communities, and those in the London region.

In conclusion, practices themselves should be cautious about further reducing appointments that are with GPs in any mode or are face to face with any healthcare professional, and should aim to provide more continuity to patients who want it.^34^ Until more evidence becomes available about the comparative impacts of transactional and relationship-based care on patient behaviours, outcomes, and use of services, policymakers should pause strategies that undermine the relationship model.

## Data

The dataset is available at: https://zenodo.org/records/16925509.
